# The Antibacterial and Antibiofilm Activity of Telithromycin Against *Enterococcus* spp. Isolated From Patients in China

**DOI:** 10.3389/fmicb.2020.616797

**Published:** 2021-01-14

**Authors:** Yanpeng Xiong, Junwen Chen, Xiang Sun, Guangjian Xu, Peiyu Li, Qiwen Deng, Zhijian Yu, Zhong Chen, Jinxin Zheng

**Affiliations:** ^1^Department of Infectious Diseases and Shenzhen Key Laboratory for Endogenous Infections, Shenzhen Nanshan People’s Hospital, Shenzhen University of School Medicine, Shenzhen, China; ^2^Quality Control Center of Hospital Infection Management of Shenzhen, Shenzhen Nanshan People’s Hospital of Guangdong Medical University, Shenzhen, China

**Keywords:** telithromycin, *Enterococcus faecalis*, *Enterococcus faecium*, erythromycin-resistance, MIC, MLST, biofilm

## Abstract

Telithromycin has been reported to possess robust *in vitro* antibacterial activity against many species of gram-positive bacteria, and telithromycin is also effective against *Staphylococcus aureus* biofilms. However, the *in vitro* antimicrobial susceptibility of telithromycin against clinical enterococci isolates in China is rarely reported and the impacts of telithromycin on the biofilm formation and eradication of enterococci remain elusive. Therefore, this study aimed to explore the inhibitory effects of telithromycin on planktonic cells and biofilms of *Enterococcus* strains. A total of 280 *Enterococcus faecalis* and 122 *Enterococcus faecium* isolates were collected from individual inpatients in China. The 50% minimum inhibitory concentration (MIC_50_) values of telithromycin against the *E. faecalis* and *E. faecium* strains carrying erythromycin-resistant methylase (*erm*) genes such as the *ermA*, *ermB*, or *ermC*, were 2 and 4 μg/mL, respectively. In addition, these isolates were typed using multilocus sequence typing (MLST) based on housekeeping genes. The predominant sequence types (STs) of *E. faecalis* were ST16, ST30, and ST179, and the main STs of *E. faecium* isolates were ST18, ST78, and ST80. Among these major STs, 87.1% (135/158) of *E. faecalis* and 80.4% (41/51) of *E. faecium* carried *erm* genes. Furthermore, at the subinhibitory concentrations (1/4 and 1/8 × MIC) of telithromycin, the biofilm formation of 16 *E. faecalis* isolates were inhibited by approximately 35%. Moreover, treatment with 8 × MIC of telithromycin or ampicillin led to an almost 40% reduction in the established biofilms of *E. faecalis* isolates, whereas vancomycin or linezolid with 8 × MIC had minimal effects. The combination of telithromycin and ampicillin resulted in an almost 70% reduction in the established biofilms of *E. faecalis*. In conclusion, these results revealed that telithromycin significantly decreased the planktonic cells of both *E. faecalis* and *E. faecium*. In addition, the data further demonstrated that telithromycin has the robust ability to inhibit *E. faecalis* biofilms and the combination of telithromycin and ampicillin improved antibiofilm activity. These *in vitro* antibacterial and antibiofilm activities suggest that telithromycin could be a potential candidate for the treatment of enterococcal infections.

## Introduction

Enterococci are gram-positive cocci which are commonly found in the gastrointestinal tracts of nearly all land animals, including humans (Fiore et al., 2019). Although a core member of the microbiome, enterococci are capable of resulting in various infectious diseases, such as urinary tract infections, wound infections, intra-abdominal and pelvic regions infections, and bloodstream infections (Murray and Weinstock, 1999; Richards et al., 2000; Mohamed and Huang, 2007). Enterococci are now the third most common nosocomial pathogen. Statistics showed that enterococci caused almost 15% of hospital-acquired infections in the United States between 2011 and 2014 (Weiner et al., 2016), an increase of 12% from 2006 to 2007 (Hidron et al., 2008). In addition, enterococci are also responsible for 5–20% of cases of infective endocarditis (Megran, 1992). *Enterococcus faecalis*, the most common species of *Enterococcus* in the clinical setting, causes 85 to 90% of human enterococcal infections, while *Enterococcus faecium* is responsible for 5 to 10% of the remainder (Jett et al., 1994; Jones et al., 2004). Because *E. faecalis* and *E. faecium* usually carry a range of intrinsic and acquired resistance genes, these *Enterococcus* strains are frequently resistant to many commonly used antibiotics, such as glycopeptides (vancomycin and teicoplanin), beta-lactams (ampicillin, penicillin), aminoglycoside (gentamicin or streptomycin), and macrolides (van Harten et al., 2017; Ch’ng et al., 2019). Of note, the widespread emergence of vancomycin-resistant enterococci (VRE) has caused further concern due to the high mortality rate (Eliopoulos, 1997). In 2013, almost 70% of clinical *E. faecium* isolates in the United States displayed vancomycin resistance, while this was up to 20% in Europe (Mendes et al., 2016). In contrast, *E. faecalis* isolates are less frequently resistant to vancomycin (<10%) (Sievert et al., 2013).

Besides antibiotic resistance, enterococci are also known for their ability to form biofilms which is a population of cells growing on a surface and surrounded by a matrix of macromolecules like polysaccharides, proteins, lipids, and extracellular DNA (Jakubovics and Burgess, 2015). The term biofilm was introduced into medicine in 1982 by Costerton as *Staphylococcus aureus* biofilms were observed on a cardiac pacemaker lead (Marrie et al., 1982). Bacteria that are not innately resistant to antibiotics can also become resistant by forming persistent biofilms that lead to chronic infections (Lebeaux et al., 2014). In today’s healthcare environment, the diversity of biofilm-associated infections has risen with time, it is suggested that biofilms are present in more than 65% of all bacterial infections (Costerton et al., 1999; Lewis, 2001). Especially, due to a sharp increase in the number of patients receiving implanted medical devices in recent years, the rates of infection are 40% for ventricular assist devices, 10% for ventricular shunts, and 4% for mechanical heart valves, pacemakers, and defibrillators (Darouiche, 2004). Biofilms serve as a new nidus for bacterial dissemination and as a reservoir for antimicrobials resistant genes. Additionally, biofilms protect bacteria from detergent solutions, antimicrobial agents, environmental stress, and effectively make bacteria 10 to 1000-fold more resistant to antibiotic treatment, making their eradication extremely difficult (Lewis, 2001). Therefore, biofilm infections are becoming increasingly difficult to effectively treat due to the decreasing efficacy of antibiotics (Paganelli et al., 2012; van Harten et al., 2017). Enterococci frequently cause biofilm-associated infections such as catheter-related bloodstream infections, urinary tract infections, and infective endocarditis (Arias and Murray, 2012). Among *Enterococcus* species, *E. faecalis* isolates usually have a higher capacity of producing biofilms than *E. faecium* isolates and the prevalence of *E. faecalis* biofilms varies in different regions (Mohamed and Huang, 2007). For example, in Sardinia, biofilm production was identified among 87% of *E. faecalis* clinical isolates and 16% of *E. faecium* clinical isolates (Duprè et al., 2003). In Rome, 80% of *E. faecalis* and 48% of *E. faecium* isolates from infected patients were able to form biofilms (Baldassarri et al., 2001). Other study showed similar results and indicated that *E. faecalis* (95%) isolates produced biofilms more often than *E. faecium* (29%) (Di Rosa et al., 2006). In contrast with planktonic cells, biofilm-embedded enterococci are involved in multidrug resistance and may even be untreatable with conventional antibiotics (Lewis, 2001). Therefore, there is an urgent need to identify novel treatments for enterococcal infections.

Telithromycin (HMR 3647), a semi-synthetic derivative of erythromycin, belongs to a new chemical class of antibiotics called ketolides that have been added to the macrolide-lincosamide-streptogramin class of antibiotics (Douthwaite and Champney, 2001). Telithromycin has been approved by the U.S. Food and Drug Administration (FDA) to treat several infectious diseases, such as community-acquired pneumonia, acute exacerbations of chronic bronchitis, and acute maxillary sinusitis (Nguyen and Chung, 2005). The antimicrobial action of telithromycin, and probably that of the other 14-member-ring macrolides, is compounded by binding to 23S rRNA and blocking protein synthesis at the early stages (Menninger, 1995). Macrolides resistance is dominantly explained by the prevalence of *erm* genes in a wide spectrum of gram-positive bacteria (Westh et al., 1995). *erm* gene classes can encode a series of methyltransferase that specifically methylate the N-6 position of adenosine 2058 (A2058) or neighboring nucleotides in domain V of 23S rRNA within the large ribosomal subunit (Weisblum, 1995; Vester and Douthwaite, 2001), this prevents interaction with macrolides. In addition, erythromycin-resistant enterococci often exhibit macrolide-lincosamide-streptogramin B (MLSB) antibiotic resistance phenotypes due to the presence of *erm* genes. However, compared with MLSB antibiotics like erythromycin, telithromycin has an additional target site at position A752 in domain II of 23S rRNA. U747 methylation promotes G748 methylation, resulting in the increased binding of telithromycin to ribosomes and enhanced telithromycin susceptibility (Shoji et al., 2015). Telithromycin has been found to conquer *erm*-mediated resistance in *S. pneumonia* and *S. aureus* (Van Laethem and Sternon, 2003; Sun et al., 2018), and it also showed less efficacy against erythromycin-resistant *Enterococcus* isolates from different regions (TEL, MIC ≥ 4 μg/mL) (Singh et al., 2000; Min et al., 2011). However, the antimicrobial impacts of telithromycin on *Enterococcus* carrying *erm* genes remain less clear in China. Moreover, a previous study reported that telithromycin exhibited effective antibiofilm activity against *S. aureus in vitro* (Zheng et al., 2020). Therefore, the effect of telithromycin on *Enterococcus* biofilms should be further investigated.

In this study, the *in vitro* antibacterial activity of telithromycin was tested against enterococcal clinical isolates from inpatients in Shenzhen Nanshan People’s Hospital, and then the antibacterial activity of telithromycin was further compared with that of other antimicrobials. In addition, the multilocus sequence types (MLSTs) and *erm* genes expression in enterococci were detected by PCR assay. Moreover, the effect of telithromycin on the biofilms of *E. faecalis* isolates was further explored.

## Materials and Methods

### Bacterial Isolates and Growth Conditions

A total of 280 *E. faecalis* and 122 *E. faecium* strains were isolated from individual patients at Shenzhen Nanshan People’s Hospital from 2011 to 2015. Bacterial isolates were determined by standard methods using a VITEK 2 system (Biomérieux, Marcy l’Etoile, France). *E. faecalis* ATCC 29212 and OG1RF (ATCC 47077) were tested as quality control strains. The isolates were cultured overnight in tryptic soy broth (TSB) (Oxoid, Basingstoke, United Kingdom) at 37°C with a shaker at 220 rpm. Telithromycin (TEL), erythromycin (ERY), ampicillin (AMP), vancomycin (VAN), tetracycline (TET), doxycycline (DOX), minocycline (MIN), ciprofloxacin (CIP), nitrofurantoin (NIT), linezolid (LZD), tedizolid (TED), and tetracycline (TEC) were purchased from MCE (Princeton, NJ, United States).

### Antimicrobials Susceptibility Testing

Antimicrobial susceptibilities of *Enterococcus* to several clinical antibiotics, including TEL, ERY, AMP, VAN, TET, DOX, MIN, CIP, NIT, LZD, TED, and TEC were tested with the VITEK 2 system. The MICs of AMP, VAN, TEL, and ERY were determined by the broth macrodilution method in cation-adjusted Mueller-Hinton broth (CAMHB) according to the 2019 Clinical and Laboratory Standards Institute (CLSI) guidelines^1^. The isolates were cultured overnight in TSB at 37°C with a shaker at 220 rpm, then the strains were diluted at 1:200 [2.0–3.0 × 10^7^ colony-forming units (CFU) ml^–1^], and inoculated into 96 polystyrene microtiter plates with 200 μL of CAMHB containing the indicated concentrations of antibiotics. As the MIC breakpoint of telithromycin against enterococci has not been established, the MIC value of telithromycin against *S. aureus* (≤1, 2, ≥4 μg/mL) was based on the 2019 CLSI guidelines. Four MIC levels were thus employed for telithromycin in the antimicrobial susceptibility analysis (≤1, 2, 4, ≥8 μg/mL). The used concentrations of indicated antibiotics are given in the figure legends and Supplementary Table 4. All experiments were performed at least three times.

### Detection of ERY Resistance Genes

DNA was extracted from all clinical enterococcal isolates with lysis buffer as templates for PCR according to the manufacturer’s instructions (Takara Bio Inc., Japan). As described previously (Bai et al., 2018), PCR analysis was performed to detect *ermA*, *ermB*, and *ermC* genes, and the primers used for PCR amplification were as follows:

*ermA*: sense: 5′- TCTAAAAAGCATGTAAAAGAAA-3′ and antisense: 5′- CGATACTTTTTGTAGTCCTTC-3′; *ermB*: sense: 5′-CCGTTTACGAAATTGGAACAGGTAAAGGGC-3′ and antisense: 5′-GAATCGAGACTTGAGTGTGC-3′; *ermC*: sense: 5′-GCTAATATTGTTTAAATCGTCAATTCC-3′ and antisense: 5′- GGATCAGGAAAAGGACATTTTAC -3′.

### Multilocus Sequence Type

The genotypes of the enterococcal isolates were analyzed by MLST. Seven pairs of housekeeping genes: *gdh, gyd, pstS, gki, aroE, xpt*, and *yqiL* for *E. faecalis*, and *atpA, ddl, gdh, purK, gyd, pstS*, and *adk* for *E. faecium* were amplified by PCR. As previously reported (Zheng et al., 2017), the purified PCR products were sequenced, and the results were submitted to the MLST database^2^ for comparison, and the sequence types (STs) of enterococci were determined. The primers used for PCR amplification are listed in Supplementary Tables 5, 6.

### Inhibition and Eradication of *E. faecalis* Biofilms

A detailed protocol has been previously reported (Zheng et al., 2020). As for the inhibition experiments, *E. faecalis* isolates were cultured overnight in TSB at 37°C with a shaker at 220 rpm, then the strains were diluted at 1:200 [2.0–3.0 × 10^7^ colony-forming units (CFU) ml^–1^], and inoculated into 96 polystyrene microtiter plates with 200 μL of TSBG (TSB with 0.5% glucose) containing the indicated concentration of antibiotics. TSBG without antimicrobials was used as an untreated control. After incubation for 24 h, the biofilm biomasses were washed three times with ddH_2_O before and after crystal violet staining, the stained biofilms were detected by optical density (OD_570_). Solithromycin was used as a positive control (Wang et al., 2020). As for the eradication assays of the established biofilms, *E. faecalis* isolates were cultivated in tryptic TSB medium at 37°C for 24 h to form matured biofilms, then they were treated with antimicrobials (8 × MIC) for 48 h with the medium replaced daily. TSBG without antimicrobials was used as an untreated control. Biofilm biomasses were stained and then detected. The used concentrations of indicated antibiotics are given in the figure legends and Supplementary Table 4. All data are representative of three independent experiments.

### Detecting the Adherent Cells in the Established Biofilms

The adherent cells in the established biofilms of *E. faecalis* were identified by the CFU numbers as described previously (Zheng et al., 2019). Briefly, the *E. faecalis* isolates were inoculated into 24 polystyrene microtiter plates with TSBG and formed mature biofilms after 24 h of static incubation. The supernatant was discarded and plates were washed, fresh TSBG containing antimicrobials was added, and TSBG without antimicrobials was used as an untreated control. After 48 h of static incubation with the medium being replaced daily, the supernatant was discarded and the remaining adherent cells in the established biofilms were collected by scratching the wall of the wells with a flat end toothpick. Finally, the bacteria were centrifuged and the numbers of CFU were determined. The used concentrations of indicated antibiotics were given in the figure legends and Supplementary Table 4. All data are representative of three independent experiments.

### Time-Kill Curve Assay

Two *E. faecalis* 16C3 and 16C6 isolates were cultured in TSB at 37°C for 16 h, then diluted 200 times with TSB and antibiotics were added to make the final concentrations at 4 × MIC. A colony count was performed after 0, 1, 3, and 24 h. Data are representative of three independent experiments.

### Statistical Analysis

The SPSS software (version 19.0) and GraphPad Prism software (version 5.0) were used for statistical analysis. For multiple comparisons, one-way analysis of variance (ANOVA) and a *post hoc*-Dunnett test were applied to analyze the data. All experiments were repeated at least three times. Sample size, n, for each experiment is given in the figure legends. Results are shown as mean ± SEM. Value differences were considered significant when ^∗^*p* < 0.05 (not significant *p* > 0.05, ^∗∗^*p* < 0.01, ^∗∗∗^*p* < 0.001).

## Results

### *In vitro* Antibacterial Activity of Telithromycin Against Enterococci Isolates

*Enterococcus faecalis* isolates (*n* = 280) and *E. faecium* isolates (*n* = 122) were retrospectively collected from different clinical specimens in China, including urine, wound secretions, blood, bile, phlegm, and other sources (Supplementary Figures 1A,B). The *in vitro* antibacterial activity of telithromycin against clinical isolates of *E. faecalis* and *E. faecium* are summarized in Tables 1, 2. As expected, these clinical isolates of enterococci showed a high frequency of resistance to erythromycin (ERY, MIC ≥ 8 μg/mL) and some commonly used tetracycline antibiotics, including tetracycline (TET, MIC ≥ 16 μg/mL), doxycycline (DOX, MIC ≥ 16 μg/mL), and minocycline (MIN, MIC ≥ 16 μg/mL), whereas they remained highly susceptible to vancomycin (VAN, MIC ≤ 4 μg/mL), nitrofurantoin (NIT, MIC ≤ 32 μg/mL), and linezolid (LZD, MIC ≤ 2 μg/mL).

**TABLE 1:** *In vitro* antibacterial activity of TEL compared with that of various antibiotics against *E. faecalis* isolates.

Antibiotic	No. isolates	Resistance rate (%)	MIC (μg/mL) breakpoints	NO.	TEL MIC (μg/mL)						ERY MIC (μg/mL)			
					≤0.5	1	2	4	≥8	MIC_50_/MIC_90_	≤0.5	1–4	≥8	MIC_50_/MIC_90_
Total	280	–	–	–	116	7	27	97	33	2/8	10	58	212	>256/>256
Ampicillin	275	0.4	≤8	274	130	6	26	83	29	2/8	8	55	211	>256/>256
			≥16	1	0	0	0	1	0	4/4	0	0	1	–
Vancomycin	280	0	≤4	278	115	7	27	96	33	2/8	10	58	210	>256/>256
			8–16	2	1	0	0	1	0	0.125/4	0	0	2	>256/>256
			≥32	0	0	0	0	0	0	–	0	0	0	–
Tetracycline	276	83.7	≤4	39	33	3	1	2	0	0.06/1	7	17	15	4/>256
			8	6	3	0	0	2	1	0.06/4	1	1	4	128/>256
			≥16	231	79	4	25	91	32	4/8	2	39	190	>256/>256
Doxycycline	280	78.9	≤4	40	36	1	2	1	0	0.06/0.125	7	19	14	2/>256
			8	19	7	2	3	6	1	2/4	1	2	16	128/>256
			≥16	221	73	4	22	90	32	4/8	2	37	182	>256/>256
Minocycline	280	73.2	≤4	43	37	2	2	2	0	0.06/2	7	21	15	2/>256
			8	32	11	0	9	9	3	2/4	1	5	26	>256/>256
			≥16	205	68	5	16	86	30	4/8	2	32	171	>256/>256
Ciprofloxacin	252	26.6	≤1	151	68	3	11	51	18	2/8	5	25	121	>256/>256
			2	34	22	1	2	8	1	0.125/4	1	12	21	128/>256
			≥4	67	24	3	14	20	6	2/8	2	12	53	>256/>256
Nitrofurantoin	254	1.2	≤32	247	102	6	26	84	29	2/8	9	52	186	>256/>256
			64	4	2	0	0	2	0	0.5/4	0	0	4	>256/>256
			≥128	3	3	0	0	0	0	0.03/0.25	0	2	1	4/>128
Linezolid	280	5.4	≤2	214	87	6	21	75	25	2/8	9	44	161	>256/>256
			4	51	23	1	6	17	4	2/4	1	11	39	>256/>256
			≥8	15	6	0	0	5	4	4/8	0	3	12	>256/>256

*MIC, minimum inhibitory concentration; TEL, telithromycin; ERY, erythromycin; MIC_50_/MIC_90_, the MIC values for 50% or 90% of bacterial growth inhibition.*

**TABLE 2:** *In vitro* antibacterial activity of TEL compared with that of various antibiotics against *E. faecium* isolates.

Antibiotic	No. isolates	Resistance rate (%)	MIC (μg/mL) breakpoints	No.	TEL MIC (μg/mL)						ERY MIC (μg/mL)			
					≤0.5	1	2	4	≥8	MIC_50_/MIC_90_	≤0.5	1–4	≥8	MIC_50_/MIC_90_
Total	122	–	–	–	25	2	12	34	49	4/8	6	12	104	>256/>256
Ampicillin	112	87.5	≤8	14	11	0	0	3	0	0.06/4	1	6	7	8/>128
			≥16	98	13	2	12	29	42	4/8	5	6	87	>128/>256
Vancomycin	120	0.0	≤4	116	23	2	12	31	48	4/8	6	11	99	128/>256
			8–16	4	2	0	0	2	0	0.06/4	0	1	3	8/>256
			≥32	0	0	0	0	0	0	–	0	0	0	–
Teicoplanin	115	0.9	≤8	114	24	2	12	33	43	4/8	6	12	96	>128/>256
			16	0	0	0	0	0	0	–	0	0	0	–
			≥32	1	0	0	0	0	1	8	0	0	1	>256/>256
Tetracycline	119	46.2	≤4	45	11	1	1	16	16	4/8	1	6	38	>128/>256
			8	19	3	0	1	3	12	8/8	2	0	17	>256/>256
			≥16	55	10	1	10	14	20	4/8	3	5	47	>256/>256
Doxycycline	120	38.3	≤4	62	15	1	4	22	20	4/8	4	8	50	>128/>256
			8	12	1	0	4	1	6	4/8	0	0	12	>256/>256
			≥16	46	9	1	4	8	24	8/8	2	4	40	>128/>256
Minocycline	120	27.5	≤4	66	16	1	4	22	23	4/8	4	8	54	>128/>256
			8	21	3	1	6	4	7	2/8	1	1	19	>256/>256
			≥16	33	6	0	2	7	18	8/8	1	3	29	>128/>256
Ciprofloxacin	113	9.7	≤1	29	10	1	7	8	3	2/4	1	5	23	>256/>256
			2	4	1	0	0	1	2	4/8	1	0	3	>256/>256
			≥4	80	13	1	5	24	37	4/8	3	7	70	>128/>256
Nitrofurantoin	114	57.0	≤32	11	2	0	3	2	4	4/8	1	1	9	>256/>256
			64	38	6	1	5	12	14	4/8	1	4	33	>256/>256
			≥128	65	16	1	4	18	26	4/8	4	7	54	>128/>256
Linezolid	122	2.4	≤2	116	22	2	12	32	48	4/8	6	10	100	>128/>256
			4	3	2	0	0	1	0	0.06/4	0	1	2	8/>256
			≥8	3	1	0	0	1	1	4/8	0	1	2	>256/>256

*MIC, minimum inhibitory concentration; TEL, telithromycin; ERY, erythromycin; MIC_50_/MIC_90_, the MIC values for 50% or 90% of bacterial growth inhibition.*

In addition, as shown in Tables 1, 2, both *E. faecalis* and *E. faecium* isolates had a high MIC_50_/MIC_90_ (the MIC values for 50% or 90% of bacterial growth inhibition) of ERY (>256/>256 μg/mL). However, the MIC_50_/MIC_90_ values of telithromycin against the *E. faecalis* and *E. faecium* strains were 2/8 μg/mL and 4/8 μg/mL, respectively. Moreover, 75.7% (212/280) of *E. faecalis* isolates were shown with ERY MIC ≥ 8 μg/mL, whereas only 11.8% (33/280) of *E. faecalis* isolates were shown with telithromycin MIC ≥ 8 μg/mL (Table 1). Similarly, among 122 *E. faecium* isolates, 85.2% (104/122) of strains had a high resistance to ERY (MIC ≥ 8 μg/mL), whereas 40% (49/122) of strains were shown with telithromycin MIC ≥ 8 μg/mL (Table 2), suggesting that *Enterococcus* isolates were more susceptible to telithromycin than ERY. Interestingly, it was found that there was a high susceptibility rate of 99.6% (274/275) for *E. faecalis* isolates toward ampicillin (MIC ≤ 8 μg/mL), whereas there was a high resistant rate of 87.5% (98/112) for *E. faecium* isolates to ampicillin (MIC ≥ 16 μg/mL). These results indicate that the antibacterial activity of telithromycin against *Enterococcus* was better than that of ERY.

### Telithromycin Against the *Enterococcus* Clinical Isolates Harboring *erm* Genes

Next, this study further examined the effects of *E. faecalis* and *E. faecium* isolates carrying *erm* genes on the sensitivity of telithromycin. At first, the presence of *ermA*, *ermB*, or *ermC* in *Enterococcus* isolates was detected by PCR assays. As shown in Table 3, the rates of *E. faecalis* strains harboring *ermA* and *ermB* genes were 3.9 and 67.1%, respectively. However, there were no *ermC*-positive *E. faecalis* isolates. Additionally, among 122 *E. faecium* isolates, the rates of *E. faecium* isolates carrying *ermA*, *ermB*, and *ermC* gene were 9.8, 32.8, and 45.9%, respectively (Table 4). Furthermore, telithromycin MIC_50_/MIC_90_ values of *ermA* and *ermB*-positive *E. faecalis* strains or *E. faecium* strains both were 4/8 μg/mL, and *ermC*-positive *E. faecium* strains had a telithromycin MIC_50_/MIC_90_ of 8/8 μg/mL (Tables 3, 4). As mentioned, the MIC_50_/MIC_90_ values of telithromycin against *E. faecalis* and *E. faecium* strains were 2/8 and 4/8 μg/mL, respectively (Tables 1, 2), suggesting that the presence of *erm* genes slight impacted telithromycin susceptibility in the *Enterococcus* isolates.

**TABLE 3:** *In vitro* activity of TEL against *E. faecalis* isolates with ERY-specific resistant genes.

Erythromycin resistance genes		No. (%)	TEL MIC (μg/mL)						ERY MIC (μg/mL)			
			≤0.5	1	2	4	≥8	MIC_50_/MIC_90_	≤0.5	1–4	≥8	MIC_50_/MIC_90_
Total		280	116	7	27	97	33	2/8	10	58	212	>256/>256
*ermA*	+	11 (3.9)	1	0	0	6	4	4/8	0	0	11	>256/>256
	−	269 (96.1)	115	7	27	91	29	2/8	10	58	201	>256/>256
*ermB*	+	188 (67.1)	39	4	23	92	30	4/8	1	8	179	>256/>256
	−	92 (32.9)	77	3	4	5	3	0.06/2	9	50	33	2/>256
*ermC*	+	0 (0)	0	0	0	0	0	–	0	0	0	–
	−	280 (100)	116	7	27	97	33	2/8	10	58	212	>256/>256

*MIC, minimum inhibitory concentration; TEL, telithromycin; ERY, erythromycin; +, positive; −, negative; MIC_50_/MIC_90_, the MIC values for 50% or 90% of bacterial growth inhibition.*

**TABLE 4:** *In vitro* activity of TEL against *E. faecium* isolates with ERY-specific resistant genes.

			No. (%)	TEL MIC (μg/mL)						ERY MIC (μg/mL)			
Erythromycin resistance genes			≤0.5	1	2	4	≥8	MIC_50_/MIC_90_	≤0.5	1–4	≥8	MIC_50_/MIC_90_
Total		122	25	2	12	34	49	4/8	6	12	104	≥0.25–256
*ermA*	+	12 (9.8)	2	0	2	3	5	4/8	0	2	10	128/>256
	−	110 (90.2)	23	2	10	31	44	4/8	6	10	94	>128/>256
*ermB*	+	40 (32.8)	2	0	3	21	14	4/8	0	2	38	128/>256
	−	82 (67.2)	23	2	9	13	35	4/8	6	10	66	>128/>256
*ermC*	+	56 (45.9)	5	2	7	10	32	8/8	0	1	55	>256/>256
	−	66 (54.1)	20	0	5	24	17	4/8	6	11	49	>128/>256

*MIC, minimum inhibitory concentration; TEL, telithromycin; ERY, erythromycin; +, positive; −, negative; MIC_50_/MIC_90_, the MIC values for 50% or 90% of bacterial growth inhibition.*

### Relationship Between Telithromycin MICs Distribution and ST Clonality

Subsequently, MLST was performed to determine the ST distribution of *E. faecalis* and *E. faecium* isolates. As shown in Supplementary Tables 1, 2, ST16, ST30, and ST179 were the predominant STs in 44 STs detected from *E. faecalis* isolates. In total, 25 STs were identified in *E. faecium* isolates, the main STs were ST18, ST78, and ST80. In addition, the relationships between telithromycin and ERY MICs distributions in the predominant ST isolates are shown in Tables 5, 6. The data indicated that *E. faecalis* with telithromycin MIC ≤ 1, 2, 4, ≥8 μg/mL accounted for 26.6% (42/158), 9.5% (15/158), 47.5% (75/158), 16.4% (26/158) and that the rates of *E. faecium* isolates with telithromycin MIC ≤ 1, 2, 4, ≥8 μg/mL were 15.7% (8/51), 2% (1/51), 23.5% (12/51), 58.8% (30/51), respectively. However, the ERY-resistant rates of *E. faecalis* and *E. faecium* isolates with the top three STs reached 98.7% (156/158) and 94.1% (48/51), respectively. Moreover, among the predominant STs isolates, 87.1% (135/158) of *E. faecalis* and 80.4% (41/51) of *E. faecium* were shown alongside the *erm* genes carriage (Tables 5, 6), demonstrating clonal clustering toward these predominant STs.

**TABLE 5:** TEL MIC values of predominant STs in *E. faecalis* isolates with the *ermB* gene.

ST	No. (%)	TEL MIC (μg/mL)						ERY MIC (μg/mL)				*ermB*
		≤0.5	1	2	4	≥8	MIC_50_/MIC_90_	≤0.5	1–4	≥8	MIC_50_/MIC_90_	
ST16	78 (27.9)	18	3	9	36	12	4/8	0	6	72	>256/>256	67
ST30	8 (2.9)	7	0	1	0	0	0.125/2	0	2	6	128/>256	6
ST179	72 (25.7)	14	0	5	39	14	4/8	2	7	63	>256/>256	62

*MIC, minimum inhibitory concentration; TEL, telithromycin; ERY, erythromycin; MIC_50_/MIC_90_, the MIC values for 50% or 90% of bacterial growth inhibition.*

**TABLE 6:** TEL MIC values of predominant STs in *E. faecium* isolates carrying the *ermB* and *ermC* genes.

ST	No. (%)	TEL MIC (μg/mL)						ERY MIC (μg/mL)				*ermB*	*ermC*
		≤0.5	1	2	4	≥8	MIC_50_/MIC_90_	≤0.5	1–4	≥8	MIC_50_/MIC_90_		
ST18	18 (12.3)	3	0	0	0	15	8/8	0	1	17	>256/>256	1	17
ST78	26 (21.3)	4	1	0	9	12	4/8	3	1	22	128/>256	16	3
ST80	7 (5.7)	0	0	1	3	3	4/8	0	0	7	>128/>128	4	0

*MIC, minimum inhibitory concentration; TEL, telithromycin; ERY, erythromycin; MIC_50_/MIC_90_, the MIC values for 50% or 90% of bacterial growth inhibition.*

### The Effects of Telithromycin Against Biofilm Formation and Eradication of *E. faecalis* Clinical Isolates

**FIGURE 1:**
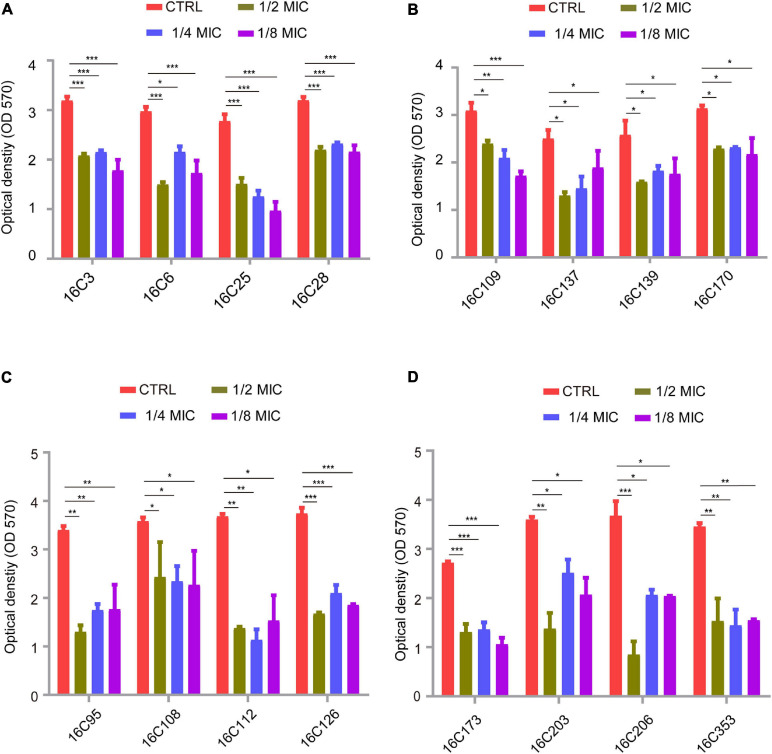
Telithromycin inhibiting biofilm formation of 16 *E. faecalis* isolates. **(A,B)** The four isolates per group were treated with TEL at 1/2 ×, 1/4 ×, and 1/8 × MICs, respectively. All of the eight isolates with the TEL MIC at 8 μg/mL. **(C,D)** The eight isolates of the two groups were treated with TEL at 1/2 ×, 1/4 ×, and 1/8 × MICs. The MICs were 0.25, 0.5, 0.125, 0.125, 0.5, 0.125, 0.25, and 0.25 μg/mL, respectively. TSBG without antimicrobials was used as an untreated control. The data were presented as the mean ± SEM (*n* = 3 experiments), one-way analysis of variance (ANOVA), and a *post hoc*-Dunnett test, *p < 0.05, **p < 0.01, ***p < 0.001, compared with the CTRL group.

Many studies have demonstrated that *E. faecalis* isolates have a higher capacity of producing biofilms than *E. faecium* (Duprè et al., 2003; Sandoe et al., 2003; Zheng et al., 2017), thus *E. faecalis* isolates are usually chosen for biofilm analysis. Among our 280 *E. faecalis* isolates, 16 *E. faecalis* isolates showed a higher biofilm-forming ability, these specific 16 strains were thus tested for biofilm formation. The isolation sites of the 16 *E. faecalis* strains are listed in Supplementary Table 3. The inhibitory effect of telithromycin on the biofilm formation of these 16 *E. faecalis* isolates was determined by crystal violet staining. The MIC values of AMP, VAN, LZD, and telithromycin against these isolates are listed in Supplementary Table 4. As shown in Figure 1, 1/2 × MIC, 1/4 × MIC, or 1/8 × MIC of telithromycin could inhibit the biofilm formation of the 16 *E. faecalis* isolates.

**FIGURE 2:**
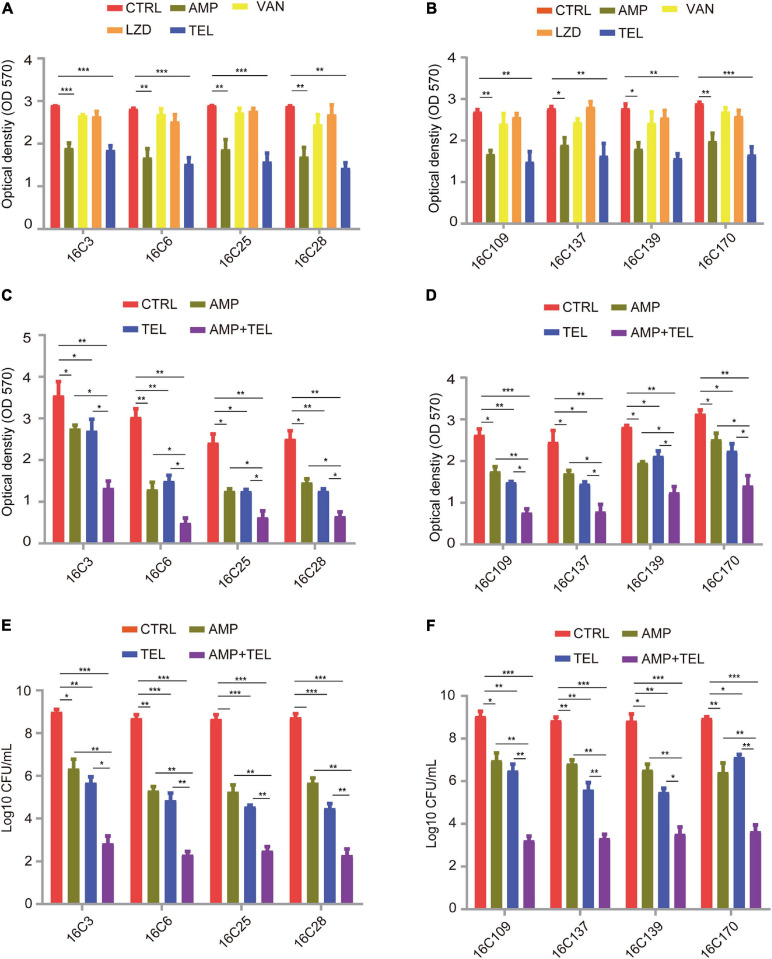
Telithromycin alone or combined with ampicillin eradicating the established biofilms of eight *E. faecalis* isolates. **(A,B)** A total of eight isolates formed mature biofilms for 24 h, then treated with AMP, VAN, LZD, or TEL at 8 × MICs for 48 h. The MICs of AMP, VAN, and TEL for these isolates were 2, 1, and 8 μg/mL, respectively. The MICs of LZD for 16C3, 16C6, 16C25, and 16C28 isolates were 4 μg/mL, the MICs for 16C109, 16C137, 16C139, and 16C170 were 2 μg/mL. **(C,D)** A total of eight isolates formed mature biofilms for 24 h, then were treated with AMP and TEL alone or TEL combined with AMP at 8 × MICs for 48 h. The MICs of AMP and TEL for these isolates were 2 and 8 μg/mL, respectively. **(E,F)** A total of eight isolates formed mature biofilms for 24 h, then were treated with AMP and TEL alone or TEL combined with AMP at 8 × MICs for 48 h. Then the adherent cells in these established biofilms were detected by the CFU numbers. The MICs of AMP and TEL for these isolates were 2 and 8 μg/mL, respectively. TSBG without antimicrobials was used as an untreated control throughout. The data were presented as the mean ± SEM (*n* = 3 experiments), one-way analysis of variance (ANOVA), and *post hoc*-Dunnett test, *p < 0.05, **p < 0.01, ***p < 0.001, compared with the CTRL group.

Finally, to dissect the effect of telithromycin on the established biofilms of *E. faecalis* isolates. Here, eight specific *E. faecalis* isolates were chosen for analysis by crystal violet staining. As shown in Figures 2A,B, the established biofilms of *E. faecali*s reduced by almost 40% after treatment with 8 × MIC of telithromycin or ampicillin, whereas 8 × MIC of vancomycin or linezolid only showed slight effects. Due to the high susceptibility rate of the *E. faecalis* isolates toward ampicillin (Table 1), the effects of telithromycin combined with ampicillin on the established biofilms of these eight *E. faecalis* isolates were further evaluated. The data showed that the combination of telithromycin and ampicillin resulted in an approximate 70% reduction in the established biofilms than with telithromycin or ampicillin alone (Figures 2C,D). Additionally, a colony-forming unit (CFU) assay was performed to quantify the viable cells of the established biofilms. Consistently, the data further confirmed that telithromycin combined with ampicillin could kill more than 70% of adherent cells in the established biofilms than telithromycin or ampicillin alone (Figures 2E,F). Therefore, these results suggest that the combination of telithromycin and ampicillin is an effective way to reduce the established biofilms in the *E. faecalis* isolates.

## Discussion

Enterococci species are commensal bacteria in the human gastrointestinal tract with the ability to cause various nosocomial infections. They not only have multiple inherent antimicrobial resistances, but are also able to acquire mutations and/or new resistance genes (Cetinkaya et al., 2000; Raza et al., 2018). Telithromycin, a novel ketolide antimicrobial agent, can be utilized for the treatment of respiratory infections. It retains its activity against most macrolide-resistant strains of *Streptococcus pneumoniae* and *Streptococcus pyogenes* (Spiers and Zervos, 2004; Wolter et al., 2008; Togami et al., 2009; Uzun et al., 2014). In addition, the efficacy of telithromycin against *Enterococcus* has been studied worldwide (Baltch et al., 2001; Bonnefoy et al., 2001; Mensa et al., 2003; Min et al., 2011), and it has been proven to be more effective against enterococci than some of first- and second-generation macrolides. For instance, telithromycin was found to be more potent against enterococci than erythromycin in a mouse peritonitis model (Singh et al., 2000). The MIC_50_/MIC_90_ of telithromycin were 8/32 μg/mL against vancomycin-resistance enterococci strains, whereas erythromycin and clarithromycin were completely inactive (Hamilton-Miller and Shah, 1998). In this study, the MIC_50_/MIC_90_ of ERY were >256/>256 μg/mL, and the resistance rate of ERY reached more than 75% in both *E. faecalis* and *E. faecium* isolates (Tables 1, 2). However, the MIC_50_/MIC_90_ of telithromycin in *E. faecalis* and *E. faecium isolates* were found to be as low as 2/8 and 4/8 μg/mL, respectively (Tables 1, 2). This study thus delineated additional evidence to support the fact that telithromycin could be a promising antimicrobial drug for use against enterococci. Of note, the majority of clinical *E. faecalis* isolates remain susceptible to β-lactams which are inhibitors of cell wall synthesis, and time-kill curve studies demonstrated that telithromycin (4 × MIC) combined with ampicillin (4 × MIC) could kill more than 100 times more planktonic cells in comparison to telithromycin or ampicillin alone in two *E. faecalis* isolates (Supplementary Figures 2A,B), suggesting that the combination of telithromycin and ampicillin could improve bactericidal activities against *E. faecalis*.

Ever since erythromycin has been clinically applied, the MLSB resistance phenotype has been widely found in erythromycin-resistant isolates of many bacteria species. For instance, Weisblum B et al. demonstrated that erythromycin-resistant *S. aureus* was related to the MLSB resistance phenotype after the clinical application of erythromycin (Weisblum and Demohn, 1969). This MLSB phenotype was determined by the *erm* genes producing erythromycin-resistant methylases, which induced methylation at specific adenosine residues on the 23S rRNA, thus leading to the resistance of the newly synthesized ribosome to MLSB antibiotics (Westh et al., 1995; Ackermann and Rodloff, 2003). Previous studies have described that the emergence of high-level erythromycin resistance narrowed the clinical application of macrolides and their application might result in a poor prognosis, more severe recurrence, and higher mortality for the treatment of bacterial infections (Min et al., 2011; Celik et al., 2014). While the chemical structure of telithromycin is derived from erythromycin, telithromycin has a low potential for drug-drug interaction, and is less likely to induce MLSB resistance than macrolides with a 14- or 15-member ring (Benes, 2004). In this study, the *ermA* and *ermB* genes were found in ERY-resistant isolates of *E. faecalis*, and *ermA*, *ermB*, and *ermC* genes were detected in *E. faecium* isolates (Tables 3, 4). However, telithromycin remained active against enterococci with *erm* genes. Recombination is involved in the genetic variation of resistance and virulence determinants, which might promote the hospital adaptation of *Enterococcus* bacteria such as *E. faecalis* and *E. faecium* (Homan et al., 2002; Ruiz-Garbajosa et al., 2006). In this study, *E. faecalis* isolates were grouped into 44 distinct STs, with the predominant STs being ST16 and ST179, which belonged to the clonal complex (CC16) (Bai et al., 2018). In addition, 25 STs were determined in *E. faecium* isolates, among which ST18 and ST78 were the main positive STs (Tables 5, 6). These results further confirmed that the presence of *erm* genes had minimal effects on the sensitivity of the predominant ST strains to telithromycin, suggesting its potential application for the treatment of some multi-resistant enterococci infections.

Evidence continues to accrue documenting the crucial role of biofilm formation in enterococcal infection (Ch’ng et al., 2019). The majority of clinical *E. faecalis* isolates are capable of forming biofilms on inanimate and living surfaces, which may promote antibiotic tolerance and reduce susceptibility to environmental influences or antimicrobial pressures (Paganelli et al., 2012; Holmberg and Rasmussen, 2016). Thus, targeting the biofilm formation of *E. faecalis* may potentially contribute to the treatment of enterococcal infections. As previously described, telithromycin could be considered as a novel inhibitor of *S. aureus* biofilms, which may be a result of the decreased expression of biofilm formation-related genes (Woo et al., 2017; Zheng et al., 2020), but there are no available data on the contribution of telithromycin susceptibility to biofilm phenotype in *E. faecalis*. The present study firstly showed that subinhibitory concentrations of telithromycin could inhibit the biofilm formation of 16 *E. faecalis* isolates (Figure 1). However, the data regarding biofilm formation inhibition does not show a dose-dependent effect. Therefore, further investigation is needed to characterize the inhibitory effect of telithromycin on the biofilm formation of *E. faecalis*.

Because biofilm prevention is not always possible, the removal of pre-existing enterococcal biofilms remains a necessity. Although the antibiofilm activity of antibiotics is likely hampered by poor penetration or slowed cell division in metabolically dormant biofilms, the use of antibiotics as first-line treatment for biofilm-associated infections is commonplace (Stewart, 2002). For example, the most recommended antibiotic treatment of endocarditis caused by *E. faecalis* involves ampicillin combined with gentamicin for 4–6 weeks (Habib et al., 2015). In this study, both telithromycin and ampicillin exhibited promising biofilm-inhibiting activity (40% reduction) in *E. faecalis* (Figures 2A,B), these results are consistent with those previously reported in the *in vitro* activities of telithromycin or ampicillin against *S. aureus* or *E. faecalis* biofilms (Di Domenico et al., 2019; Zheng et al., 2020). Notably, treatment with an 8 × MIC telithromycin/ampicillin combination exhibited a visible reduction (∼70%) in mature *E. faecalis* biofilms (Figures 2C,D) and killed 70% of adherent cells in the established biofilms (Figures 2E,F). Therefore, the combination of telithromycin with ampicillin as an anti-adherence or anti-biofilm strategy appears to be much more promising for the treatment of biofilm-associated enterococcal infections.

Accumulating evidence has revealed that there is a lower likelihood of resistance developing through the clinical use of telithromycin. For example, spontaneous resistance to telithromycin was at a low frequency *in vitro*. Telithromycin does not induce MLSB resistance and it shows low potential in selecting resistance or cross-resistance (Felmingham, 2001). In addition, pharmacodynamic studies suggested that telithromycin was generally well tolerated and a once-daily 800 mg oral dose of telithromycin maintains an effective concentration in plasma for the treatment of respiratory tract infections involving the key respiratory pathogens (Drusano, 2001; Namour et al., 2001). However, information regarding kill kinetics and post-antibiotic effects for telithromycin against enterococci is limited. Thereafter, there is inadequate clinical evidence to suggest an optimal dosage regimen for telithromycin against enterococci. The present study demonstrated the antimicrobial susceptibility and antibiofilm activity of telithromycin against *Enterococcus* isolates *in vitro*, suggesting that this compound might be useful, alone or in combination, in some difficult to treat enterococcal infections. The further results of *in vivo* studies should provide some evidence to support such a possibility.

## Conclusion

In summary, this study presents the effective antimicrobial activity of telithromycin against clinical enterococci isolates from China in comparison to that of ERY. Importantly, this study further demonstrated that telithromycin could inhibit the biofilm formation of *E. faecalis* and that telithromycin combined with ampicillin resulted in enhanced antimicrobial and antibiofilm activity. To our knowledge, this is the first study to present new insight into the antibiofilm activity of telithromycin against enterococci and provide further evidence for the potential clinical application of telithromycin/ampicillin combination for the treatment of *Enterococcus* infections.

## Data Availability Statement

The original contributions presented in the study are included in the article/Supplementary Material, further inquiries can be directed to the corresponding authors.

## Author Contributions

YX conducted the PCR analyses, MLST analysis, biofilms inhibition and eradication of *E. faecalis*, and drafted the manuscript. JC collected the bacterial isolates, performed the antimicrobials susceptibility tests, and gene manipulation. XS participated in gene manipulation, MLST analysis, and the inhibition of *E. faecalis* biofilms. GX and PL participated in the collection of bacterial isolates, MLST analysis, and the inhibition and eradication of *E. faecalis* biofilms. ZY and QD participated in the collection of bacterial isolates, and the inhibition and eradication of *E. faecalis* biofilms. ZC and JZ designed the study, analyzed the experimental data, and revised the manuscript. All authors have read and approved the manuscript.

## Conflict of Interest

The authors declare that the research was conducted in the absence of any commercial or financial relationships that could be construed as a potential conflict of interest.

## Footnotes

1 https://eucast.org/clinical_breakpoints/

2 http://efaecalis.mlst.net/ and http://efaecium.mlst.net/

## Abbreviations

AMP: ampicillin
CIP: ciprofloxacin
DOX: doxycycline
ERY: erythromycin
LZD: linezolid
MICs: minimum inhibitory concentrations
MIN: minocycline
MLST: multilocus sequence type
NIT: nitrofurantoin
PCR: polymerase chain reaction
STs: sequence types
TEC: tetracycline
TED: tedizolid
TEL: telithromycin
TET: tetracycline
VAN: vancomycin.
