# Supplementary figures and images for: The Antibacterial and Antibiofilm Activity of Telithromycin Against Enterococcus spp. Isolated From Patients in China
Source: Front Microbiol. 2021 Jan 14;11:616797. doi: 10.3389/fmicb.2020.616797 (PMC7841295; doi:10.3389/fmicb.2020.616797)

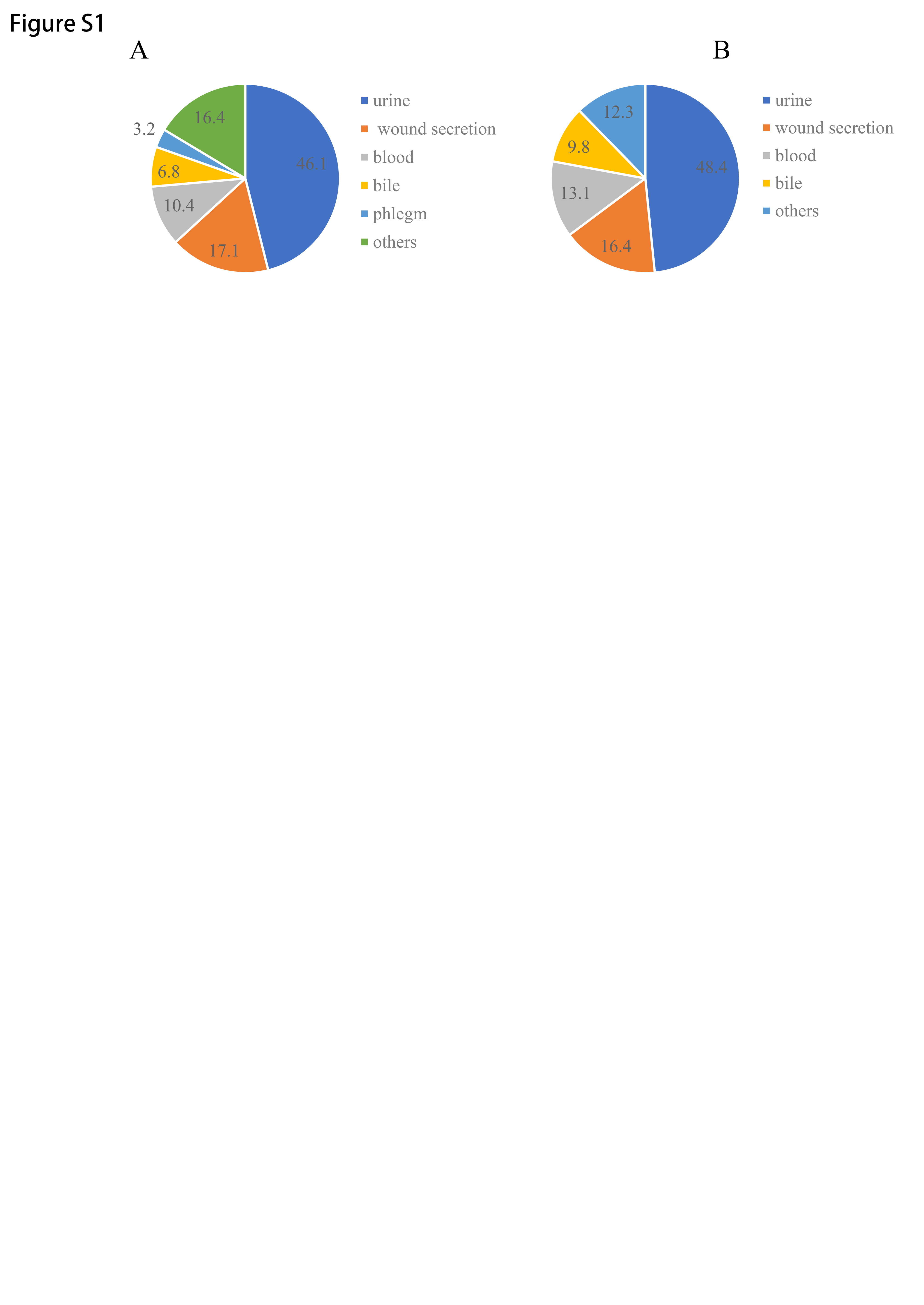

Supplement: Supplementary file 1 [file Image_1.TIF]

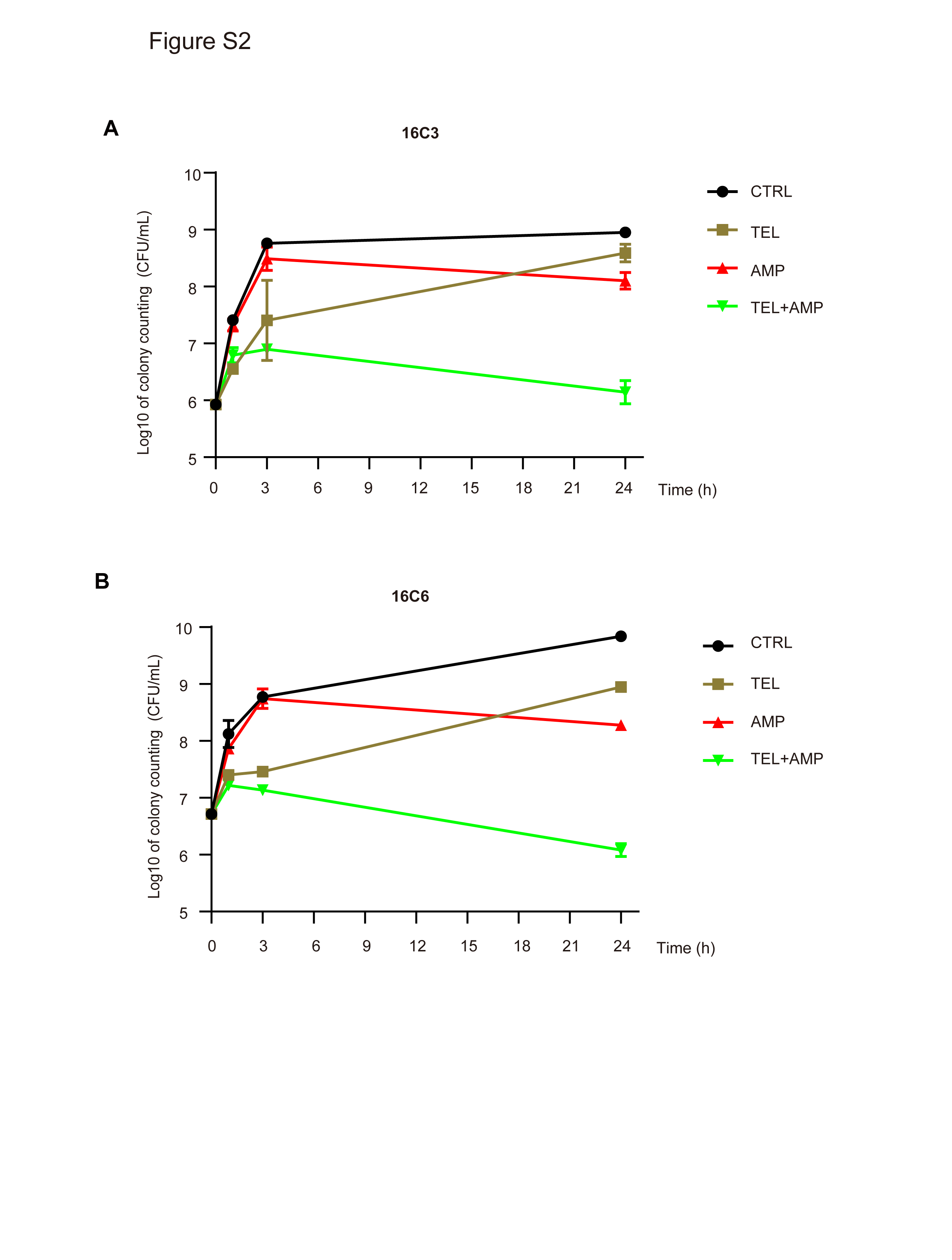

Supplement: Supplementary file 2 [file Image_2.TIF]
