# Supplementary material for: The Antibacterial and Antibiofilm Activity of Telithromycin Against Enterococcus spp. Isolated From Patients in China
Source: Front Microbiol. 2021 Jan 14;11:616797. doi: 10.3389/fmicb.2020.616797 (PMC7841295; doi:10.3389/fmicb.2020.616797)
Supplement: Supplementary file 3 [file Data_Sheet_1.docx]

**Supplementary Material**

**This file includes:**

**Supplementary Tables / Figures**

**Table S1** MICs of TEL and ERY to different STs in *E. faecalis* isolates.

**Table S2** MICs of TEL and ERY to different STs in *E. faecium* isolates.

**Table S3** The clinical sources of 16 *E. faecalis* strains

**Table S4** The 16 *E. faecalis* isolates used to form biofilms.

**Table S5** The primers used for PCR and sequence types of *E. faecalis* isolates.

**Table S6** The primers used for PCR and sequence types of *E. faecium* isolates.

**Fig. S1** **The clinical sources of enterococcal isolates**. (A) The clinical sources of *E. faecalis* isolates. (B) The clinical sources of *E. faecium* isolates. The data was displayed as a percentage (%).

**Fig. S2 The efficacy of telithromycin combined with ampicillin in the *in vitro* antibacterial activity.** (A) 16C3 and (B) 16C6 two strains treated with AMP and TEL alone or TEL combined with AMP at 4× MICs. Colony count was performed after 0 h, 1 h, 3 h and 24 h times point. The MICs of AMP and TEL for these isolates were 2 μg /mL and 8 μg /mL respectively. TSB without antimicrobials was used as an untreated control. The data presented the mean ± SEM (n=3 experiments).

**Table S1** MICs of TEL and ERY in different STs in *E. faecalis* isolates

| **ST** | **N** | **No. of isolates with each MICs (μg/mL)** | | | | | | | | | | |
| --- | --- | --- | --- | --- | --- | --- | --- | --- | --- | --- | --- | --- |
|  |  | **TEL MIC** | | | | | |  | **ERY MIC** | | | |
|  |  | ≤0.5 | 1 | 2 | 4 | ≥8 | MIC_50_ /MIC_90_ |  | ≤0.5 | 1-4 | ≥8 | MIC_50_ /MIC_90_ |
| **ST4** | 3 | 1 | 1 | 1 | 0 | 0 | 1/2 |  | 0 | 0 | 3 | >256/>256 |
| **ST6** | 5 | 1 | 1 | 0 | 2 | 1 | 4/8 |  | 0 | 1 | 4 | >256/>256 |
| **ST16** | 78 | 18 | 3 | 9 | 36 | 12 | 4/8 |  | 0 | 6 | 72 | >256/>256 |
| **ST21** | 4 | 4 | 0 | 0 | 0 | 0 | 0.0625/0.0625 |  | 1 | 2 | 1 | 1/256 |
| **ST28** | 2 | 1 | 0 | 1 | 0 | 0 | 0.25/2 |  | 0 | 0 | 2 | >256/>256 |
| **ST30** | 8 | 7 | 0 | 1 | 0 | 0 | 0.125/2 |  | 0 | 2 | 6 | 128/>256 |
| **ST40** | 4 | 4 | 0 | 0 | 0 | 0 | 0.0625/0.125 |  | 1 | 2 | 1 | 2/4 |
| **ST41** | 2 | 2 | 0 | 0 | 0 | 0 | 0.0625/0.0625 |  | 0 | 1 | 1 | 1/64 |
| **ST47** | 2 | 2 | 0 | 0 | 0 | 0 | 0.0625/0.125 |  | 0 | 1 | 1 | 2/64 |
| **ST179** | 72 | 14 | 0 | 5 | 39 | 14 | 4/8 |  | 2 | 7 | 63 | >256/>256 |
| **ST191** | 3 | 3 | 0 | 0 | 0 | 0 | 0.0625/0.0625 |  | 0 | 2 | 1 | 2/>256 |
| **ST314** | 2 | 2 | 0 | 0 | 0 | 0 | 0.0625/0.0625 |  | 0 | 2 | 0 | 2/2 |
| **ST387** | 3 | 3 | 0 | 0 | 0 | 0 | 0.0625/0.125 |  | 1 | 2 | 0 | 0.5/2 |
| **ST403** | 5 | 1 | 0 | 1 | 3 | 0 | 4/4 |  | 0 | 1 | 4 | >256/>256 |
| **ST409** | 4 | 4 | 0 | 0 | 0 | 0 | 0.25/0.5 |  | 0 | 1 | 3 | 16/>256 |
| **ST480** | 6 | 1 | 1 | 0 | 3 | 1 | 4/8 |  | 0 | 1 | 5 | >256/>256 |
| **ST506** | 2 | 1 | 0 | 0 | 0 | 1 | 0.125/8 |  | 0 | 1 | 1 | 2/128 |
| **ST541** | 5 | 3 | 0 | 0 | 2 | 0 | 0.0625/4 |  | 0 | 3 | 2 | 2/>256 |
| **NT** | 44 | 28 | 0 | 5 | 8 | 3 | 0.125/4 |  | 5 | 13 | 26 | >256/>256 |
| **Other STs** | 26 | 16 | 1 | 4 | 4 | 1 | 0.25/4 |  | 0 | 10 | 16 | 128/>256 |

Note: MIC, minimum inhibitory concentration; TEL, telithromycin; ERY, erythromycin;

**Table S2**  MICs of TEL and ERY in different STs in *E. faecium* isolates

| **ST** | **N** | **No. of isolates with each MICs (μg/mL)** | | | | | | | | | | |
| --- | --- | --- | --- | --- | --- | --- | --- | --- | --- | --- | --- | --- |
|  |  | **TEL MIC** | | | | | |  | **ERY MIC** | | | |
|  |  | ≤0.5 | 1 | 2 | 4 | ≥8 | MIC_50_ /MIC_90_ |  | ≤0.5 | 1-4 | ≥8 | MIC_50_ /MIC_90_ |
| **ST18** | 18 | 3 | 0 | 0 | 0 | 15 | 8/8 |  | 0 | 1 | 17 | >256/>256 |
| **ST78** | 26 | 4 | 1 | 0 | 9 | 12 | 4/8 |  | 3 | 1 | 22 | 128/>256 |
| **ST80** | 7 | 0 | 0 | 1 | 3 | 3 | 4/8 |  | 0 | 0 | 7 | >128/>128 |
| **ST192** | 3 | 0 | 0 | 0 | 3 | 0 | 4/4 |  | 0 | 0 | 3 | >128/>256 |
| **ST201** | 3 | 2 | 0 | 0 | 1 | 0 | 0.031/4 |  | 2 | 0 | 1 | 0.25/>256 |
| **ST202** | 2 | 0 | 0 | 0 | 0 | 2 | 0.0625/0.0625 |  | 0 | 0 | 2 | >256/>256 |
| **ST203** | 2 | 0 | 0 | 0 | 1 | 1 | 0.25/2 |  | 0 | 0 | 2 | >256/>256 |
| **ST261** | 2 | 2 | 0 | 0 | 0 | 0 | 0.0625/0.5 |  | 0 | 2 | 0 | 2/2 |
| **ST262** | 3 | 0 | 0 | 1 | 2 | 0 | 4/4 |  | 0 | 0 | 3 | >256/>256 |
| **ST323** | 5 | 0 | 0 | 0 | 1 | 4 | 8/8 |  | 0 | 0 | 5 | >256/>256 |
| **ST555** | 2 | 0 | 0 | 1 | 1 | 0 | 2/4 |  | 0 | 0 | 2 | >128/>128 |
| **NT** | 35 | 9 | 1 | 7 | 10 | 8 | 8/8 |  | 0 | 4 | 31 | >128/>256 |
| **Other STs** | 14 | 5 | 0 | 2 | 3 | 4 | 2/8 |  | 1 | 4 | 9 | >256/>256 |

Note: MIC, minimum inhibitory concentration; TEL, telithromycin; ERY, erythromycin;

**Table S3 The clinical sources of 16 *E. faecalis* strains**

| **Isolates** | **isolation site** |
| --- | --- |
| **16C3** | urine |
| **16C6** | urine |
| **16C25** | urine |
| **16C28** | urine |
| **16C109** | urine |
| **16C137** | wound secretion |
| **16C139** | urine |
| **16C170** | wound secretion |
| **16C95** | urine |
| **16C108** | wound secretion |
| **16C112** | urine |
| **16C126** | urine |
| **16C173** | bile |
| **16C203** | urine |

**Table S4** The antimicrobial susceptibilities of *E. faecalis* isolates were used to form biofilms

| **Isolates** | **MIC (μg/mL)** | | | |
| --- | --- | --- | --- | --- |
|  | **AMP** | **TEL** | **VAN** | **LZD** |
| **16C3** | 2 | 8 | 1 | 4 |
| **16C6** | 2 | 8 | 1 | 4 |
| **16C25** | 2 | 8 | 1 | 4 |
| **16C28** | 2 | 8 | 1 | 4 |
| **16C109** | 2 | 8 | 1 | 2 |
| **16C137** | 2 | 8 | 1 | 2 |
| **16C139** | 2 | 8 | 1 | 2 |
| **16C170** | 2 | 8 | 1 | 2 |
| **16C95** | 2 | 0.25 | 1 | 4 |
| **16C108** | 2 | 0.5 | 1 | 4 |
| **16C112** | 2 | 0.125 | 0.5 | 4 |
| **16C126** | 2 | 0.125 | 1 | 1 |
| **16C173** | 2 | 0.5 | 2 | 2 |
| **16C203** | 2 | 0.125 | 1 | 4 |
| **16C206** | 2 | 0.25 | 0.5 | 1 |
| **16C353** | 2 | 0.25 | 1 | 1 |

Note: MIC, minimum inhibitory concentration; AMP, ampicillin; TEL, telithromycin;

**Table S5** The primer used for PCR and sequence types of *E. faecalis* isolates

| **Primer** | **Sequence (5'-3')** |
| --- | --- |
| **gdh-sense** | GGCGCACTAAAAGATATGGT |
| **gdh-antisense** | CCAAGATTGGGCAACTTCGTCCCA |
| **gyd-sense** | CAAACTGCTTAG CTCCAATGGC |
| **gyd-antisense** | CATTTCGTTGTCATACCAAGC |
| **pstS-sense** | CGGAACAGGACTTTCGC |
| **pstS-antisense** | ATTTACATCACGTTCTACTTGC |
| **gki-sense** | GATTTTGTGGGAATTGGTATGG |
| **gki-antisense** | ACCATTAAAGCAAAATGATCGC |
| **aroE-sense** | TGGAAAACTTTACGGAGACAGC |
| **aroE-antisense** | GTCCTG TCCATTGTTCAAAAGC |
| **xpt-sense** | AAAATGATGGCCGTGTATTAGG, |
| **xpt-antisense** | AACGTCACCGTTCCTTCACTTA |
| **yqiL-sense** | CAGCTTAAGTCAAG TAAGTGCCG |
| **yqiL-antisense** | GAATATCCCTTCTGCTTGTGCT |

**Table S6** The primer pairs used for PCR and sequence types of *E. faecium* isolates

| **Primer** | **Sequence (5'-3')** |  |
| --- | --- | --- |
| **adk-sense** | TATGAACCTCATTTTAATGGG |  |
| **adk-antisense** | GTTGACTGCCAAACGATTTT |  |
| **atpA-sense** | CGGTTCATACGGAATGGCACA |  |
| **atpA-antisense** | AAGTTCACGATAAGCCACGG |  |
| **ddI-sense** | GAGACATTGAATATGCCTTAT |  |
| **ddI-antisense** | AAAAAGAAATCGCACCG |  |
| **ddh-sense** | GGCGCACTAAAAGATATGGT |  |
| **gdh-antisense** | CCAAGATTGGGCAACTTCGTCCCA |  |
| **gyd-sense** | CAAACTGCTTAGCTCCAAGGC |  |
| **gyd-antisense** | CATTTCGTTGTCATACCAAGC |  |
| **purK-sense** | CAGATTGGCACATTGAAAG |  |
| **purK-antisense** | TTCATTCACATATAGCCCG |  |
| **pstS-sense** | TTGAGCCAAGTCGAAGCTGGAG |  |
| **pstS-antisense** | CGTGATCACGTTCTACTTCC |  |
